# Electrocardiographic Changes and Their Association With Disease Severity in Adults With Sickle Cell Anemia at a Tertiary Care Center: A Cross-Sectional Study

**DOI:** 10.7759/cureus.60197

**Published:** 2024-05-13

**Authors:** I G Priyan, Preetam Wasnik, Pankaj K Kannauje, Pranita Das, Satyajit Singh, Suprava Patel

**Affiliations:** 1 General Medicine, All India Institute of Medical Sciences, Raipur, Raipur, IND; 2 Cardiology, All India Institute of Medical Sciences, Raipur, Raipur, IND; 3 Biochemistry, All India Institute of Medical Sciences, Raipur, Raipur, IND

**Keywords:** disease severity, cardiovascular manifestations, chhattisgarh, ecg, sickle cell crisis, sickle cell disease

## Abstract

Introduction

Sickle cell anemia (SCA), a severe hematological disorder, is characterized by the presence of sickle-shaped erythrocytes that obstruct capillaries and restrict blood flow. This pathophysiology not only promotes systemic complications but may also influence cardiac function. Cardiac complications are a leading cause of mortality in SCA patients, yet the specific electrocardiographic (ECG) changes associated with disease severity are not thoroughly understood. This cross-sectional study aimed to explore ECG abnormalities in adults with SCA and correlate these findings with disease severity.

Methods

An observational cross-sectional study was conducted over 18 months, from January 2022 to June 2023, among 140 SCA patients at the Sickle Cell OPD of All India Institute of Medical Sciences, Raipur, Raipur, India. Steady-state SCA (HbS >50%) patients screened by high-performance liquid chromatography were enrolled. A history, physical examination, complete blood count, and ECG were done for all cases. The disease severity score was calculated using the Adegoke and Kuti severity scores, and their association with various ECG changes was studied. The chi-square test (Fisher’s exact test, wherever applicable) was used for comparing the proportion. The correlation was done using the Pearson correlation coefficient or Spearman’s rho.

Results

Out of 140 patients, the mean age of the study participants was 26 ± 6 years. More than half of the cases (80; 57%) fall under the 18-27 age group, with a male-to-female ratio of 4:3. A total of 99 (70.7%) of the participants had mild disease, and 41 (29.3%) had moderate disease. The QT interval was significantly higher among patients with mild disease compared to those with moderate disease (p-value: <0.01). QTc dispersion and prolonged QTc interval were significantly higher among patients with moderate disease compared to mild disease (p-value <0.01, 0.04, respectively). Sinus tachycardia and right ventricular hypertrophy with p-pulmonale were significantly higher in moderate severity (p < 0.01). A significant positive correlation was observed between QTc dispersion, P-wave dispersion, and severity (r: 0.19, 0.17; p-value: 0.02, 0.04, respectively).

Conclusion

As the disease severity progressed, the ECG changes studied had a higher distribution and significance. ECG is a readily and widely accessible investigation that can be used to screen all SCA patients for early recognition of various underlying cardiac complications.

## Introduction

Sickle cell disease (SCD) falls under the category of hemoglobinopathies that include mutations in the gene encoding the beta subunit of hemoglobin. The sickle cell mutation resulting in the production of abnormal hemoglobin occurs when negatively charged glutamine is replaced by a neutral valine at the sixth position of the beta-globin chain (point mutation). This mutation is transmitted via Mendelian genetics and is inherited in an autosomal recessive fashion. A homozygous mutation leads to the severest form of SCD, i.e., sickle cell anemia (SCA), also called HBSS disease [1].

SCA is ubiquitous in the tribal belts of Chhattisgarh, Odisha, Madhya Pradesh, and Maharashtra. The frequency of sickle cell trait in India is 4.3%. In Odisha and Chhattisgarh, the frequency of SCA is approximately 9% and 10%, respectively. According to the previous study done in Chhattisgarh, sickle cell trait occurred in 9.30% and an SS phenotype in 0.21% [2].

The basic pathophysiology of SCA is based on deoxy-HbS polymerization and the production of long fibers within RBCs, resulting in a distorted sickle shape, which leads to enhanced hemolysis and sickle red cell vaso-occlusion [3].

Adults with SCA experience a variety of symptoms. The effects of recurrent hemolysis, anemia, and ischemia-reperfusion injury on multiple organs, including the heart, are responsible for these symptoms. These factors determine the severity of the illness. Recent work has shown the importance of red cell dehydration, abnormal RBC adhesion to the vascular endothelium, inflammatory events, activation of cells in the vessel, and abnormalities of nitric oxide metabolism in the pathophysiology of this multi-organ disease [3].

Diagnosis of SCA can be based on clinical background, and diagnosis can be supported by lab parameters like a complete blood count and peripheral blood smear [4,5]. The diagnosis in developing countries is mostly confirmed by high-performance liquid chromatography (HPLC) techniques, while in resourceful settings, it is also confirmed by various genetic testing techniques like PCR-based techniques, restriction fragment length polymorphism, DNA microarrays, and sequencing techniques [6-11].

As significant proportions of SCA patients survive into adulthood due to improved healthcare services, it is known that cardiovascular comorbidities contribute significantly to the complications of SCA and contribute to mortality [12].

Electrocardiography (ECG) is an affordable and relatively accessible, noninvasive procedure used to assess cardiac structure and function. Several studies have observed abnormal electrocardiograms in SCA patients. These studies have documented abnormalities such as tachycardia, left ventricular hypertrophy (LVH), left atrial fibrillation, right ventricular hypertrophy (RVH), nonspecific changes of the ST segment and T-wave, arrhythmias (such as ventricular premature complexes and atrial enlargement), prolonged corrected QT intervals, and first-degree atrioventricular block. When compared to normal controls, SCA participants also showed significantly greater mean heart rate, P-wave duration, P-wave dispersion, PR interval, QRS duration, QRS dispersion, QTc interval, and QTc dispersion [13].

These changes are observed secondary to increased cardiac output to compensate for the chronic anemia seen in SCA. There is a high output state, and the resulting cardiomegaly increases the preload. The increased preload and decreased afterload compensate for the left ventricular dysfunction and maintain a normal ejection fraction and high cardiac output. Holloman et al. reported that in the ECGs of 87 adult patients, 63 (72%) of all patients had abnormal ECGs. Nonspecific ST-T (NS ST-T) wave abnormalities 46 (53%) and QT interval prolongation 11 (12%) were frequent. A total of 10 (11%) had sinus tachycardia, and eight (80%) of those were women. Moreover, 15 of 21 (71%) patients with arrhythmias had NS ST-T abnormalities. Systemic hypertension and ECG evidence for right-sided heart disease were rare, as was the incidence of LVH by ECG [14].

Ahmed et al. reported that the estimated mean left ventricle ejection fraction was 61.29 ± 11.29% (range 20-76%) [12]. Eight (21%) patients had evidence of a hyperdynamic left ventricle (ejection fraction >70%). Left heart abnormalities included a dilated atrium in 14 (37%), a dilated ventricle in five (13%), ventricle hypertrophy in five (13%), and ventricle dysfunction in three (9%) patients. Right heart abnormalities included a dilated atrium in nine (24%), a dilated ventricle in six (16%), and ventricle dysfunction in three (9%) patients. Moreover, one of these three patients had evidence of biventricular failure, and all three patients with right ventricular dysfunction had moderate to severe pulmonary hypertension. Dosunmu et al. study showed 68 of 93 (73.1%) had abnormal ECG, while only two of 90 (2.2%) of controls had abnormal ECG. The common abnormalities observed were LVH, biventricular hypertrophy, and RVH [15].

Oguanobi et al. showed the mean heart rate, P-wave duration, P-wave dispersion, PR interval, QRS duration, QRS dispersion, QTc interval, and QTc dispersion were significantly higher in the patients than in the control group. ECG abnormalities identified by this study were LVH (75% versus 1.7%), left atrial enlargement (40% versus 0%), biventricular hypertrophy (11% versus 0%), ST-segment elevation (10% versus 0%), and increased P-wave and QTc dispersions [16,17].

The cardiovascular manifestations of SCA include cardiomegaly, cardiac failure, cardiomyopathies, diastolic dysfunction, pulmonary hypertension, etc. ECG is an affordable, relatively accessible, and noninvasive assessment method.

Different scoring systems have been used to assess the severity of the disease. Evaluation of disease severity in patients with SCA will help identify patients at higher risk for an adverse clinical course who may need more active management and monitoring. There have been several studies regarding the different ECG changes in SCA patients, and some studies have determined the disease severity based on genetic factors and phenotypic expression of the disease, such as the frequency of crises and hospital admissions, the degree of anemia, the frequency of blood transfusions, and the presence of complications. However, very little research has been done that correlates ECG changes with the disease severity of SCA.

## Materials and methods

This was a cross-sectional study conducted over 18 months, from January 2022 to June 2023, at Sickle Cell OPD, All India Institute of Medical Sciences (AIIMS), Raipur, Raipur, India. This study was approved by our institute’s ethical committee with approval number AIIMS/RPR/IEC/2022/1145.

A total of 140 SCA patients who satisfied the inclusion and exclusion criteria were enrolled in the study after obtaining informed consent. Table 1 shows the inclusion and exclusion criteria for the study.

**Table 1:** Inclusion and exclusion criteria ^*^ Steady-state: (1) Absence of any crisis in the preceding four weeks and absence of any symptom or sign attributable to acute illness. (2) No treatment with medications such as antibiotics that may affect the blood counts during the last four weeks [14]. SCA, sickle cell anemia; SCD, sickle cell disease

Inclusion criteria	Exclusion criteria
Patients aged 18-40 years suffering from SCA who are in a steady state*	Patients <18 years or >40 years
	Patients with sickle cell crises
	Patients with SCD other than homozygous type
	Patients with comorbidities having cardiac implications, such as diabetes mellitus, pregnant women, known cardiac diseases other than those secondary to SCA, hypertension, etc.

Methodology

Screening was done with HPLC reports. Only SS cases were enrolled (HbS window >50%). Steady-state SCA patients attending sickle cell OPD were recruited as per inclusion and exclusion criteria. Informed written consent for participation was obtained. A thorough clinical history and examination were done. The disease severity score was calculated for each case using the Adegoke and Kuti severity scoring system in Table 2 [18]. ECG changes were recorded manually: rate, rhythm, axis, P-wave duration, P-wave dispersion, PR interval, QRS duration, dispersion, QT interval, QTc interval, QTc dispersion, ST segment, T waves, and others. All the relevant data was stored in a Microsoft Excel master chart (Microsoft Corporation, Redmond, Washington, United States). The data was entered into a computer-based spreadsheet and cleaned. The cleaned data were analyzed using IBM SPSS Statistics for Windows, Version 25.0 (Released 2017; IBM Corp., Armonk, NY, USA). The Kolmogorov-Smirnov test was used to assess the normality of the statistical analysis comprised of calculating the mean, median, and proportions. The chi-square test (Fisher’s exact test, wherever applicable) was used for comparing the proportion. A student t-test (Mann-Whitney U test, wherever applicable) was used for comparing continuous variables. The correlation was done using the Pearson correlation coefficient or Spearman’s rho. The level of significance was taken as p < 0.05. As it was an observational cross-sectional study, data values were gathered from routine blood tests and ECG only, which were available at AIIMS Raipur. So, this study had nil risk to the participants.

**Table 2:** Adegoke and Kuti severity scoring system SCA is graded as a mild disease if the severity score is less than 8, moderate if the score is between 8 and 17, and severe if the score is greater than 17 [18]. SCA, sickle cell anemia

S.N.	Parameters	Score
1	For a number of painful episodes in the previous 12 months	
	0	0
	1	1
	2/3	2
	>3	3
2	For the number of transfusions in the previous 12 months	
	0	0
	1	1
	2/3	2
	>3	3
3	For the number of hospitalizations in the previous 12 months	
	0	0
	1	1
	2/3	2
	>3	3
4	For liver enlargement	
	<2 cm	0
	2-5 cm	1
	>5 cm	2
5	For splenic enlargement	
	<2 cm	0
	2-5 cm	1
	>5 cm	2
6	Packed cell volume	
	More than or equal to 24%	0
	18-23%	1
	<8%	2
7	WBCs	
	<11,000	0
	11,000-15,000	1
	>15,000	2
8	For the lifetime cumulative incidence of specific complications	(0 when absent)
	Cerebrovascular disease	5
	Acute coronary syndrome	3
	Pneumococcal meningitis	3
	Avascular necrosis of bone	2
	Gallstones, chronic leg ulcer, osteomyelitis, or priapism	1

## Results

A total of 140 patients were enrolled in the study. The mean age of the study participants was 26.52 ± 6.71 years. Eighty (57%) of the participants were male, and the rest (60; 43%) were female, with a male-to-female ratio of 4:3. The mean HbS% among the study participants was 66.80 ± 8.02.

**Figure 1:**
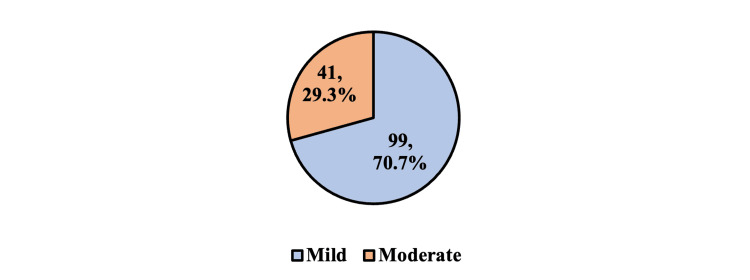
Distribution of the study participants according to disease severity

As shown in Figure 1, 99 (70.7%) of the participants had mild disease, and 41 (29.3%) had moderate disease. No severe disease study participant was found in the study.

**Figure 2:**
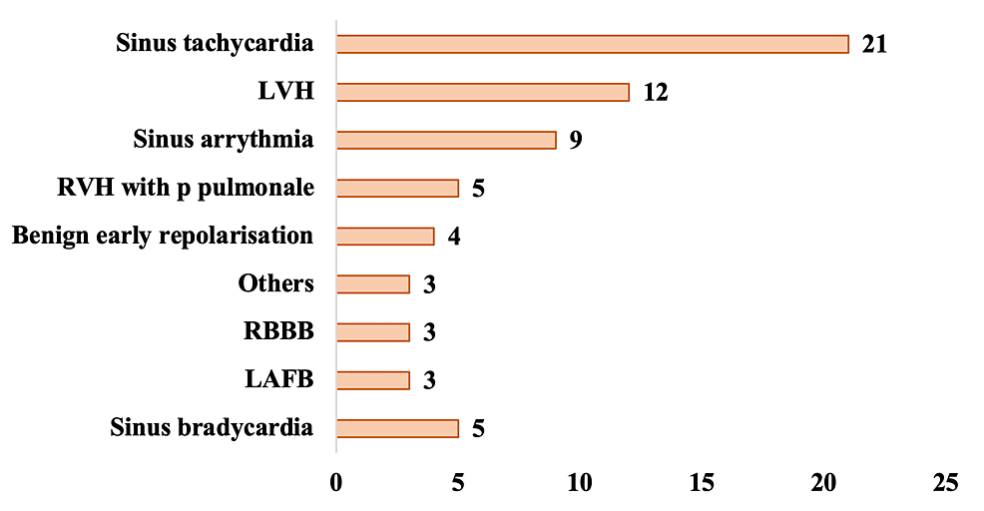
Distribution of the study participants according to ECG findings LAFB, left anterior fascicular block; LVH, left ventricular hypertrophy; RBBB, right bundle branch block; RVH, right ventricular hypertrophy

Figure 2 shows the ECG findings of the study participants. The predominant ECG finding was sinus tachycardia (21 cases; 15.7%), followed by LVH (12 cases; 8.6%), sinus arrhythmia (nine cases; 6.4%), sinus bradycardia (five cases; 3.6%), RVH with p-pulmonale (five cases; 3.6%), benign early repolarization (four cases; 2.9%), left anterior fascicular block (LAFB) (three cases; 2.1%), and right bundle branch block (RBBB) (three cases; 2.1%).

Table 3 shows the mean P-wave duration, P-wave dispersion, PR interval, QRS duration, QRS dispersion, QT interval, QTc interval, and QTc dispersion were 94.74 ± 17.72, 43.85 ± 14.32, 142.76 ± 22.29, 88.46 ± 11.83, 43.79 ± 10.5, 368.56 ± 38.41, 423.89 ± 26.86, and 40.86 ± 16.18 ms, respectively.

**Table 3:** Mean duration of ECG indices

Indices	Mean + SD
P-wave duration (ms)	94.74 ±17.72
P-wave dispersion (ms)	43.85 ± 14.32
PR interval (ms)	142.76 ± 22.29
QRS duration (ms)	88.46 ± 11.83
QRS dispersion (ms)	43.79 ±10.5
QT interval (ms)	368.56 ± 38.41
QTc interval (ms)	423.89 ± 26.86
QTc dispersion (ms)	40.86 ± 16.18

**Figure 3:**
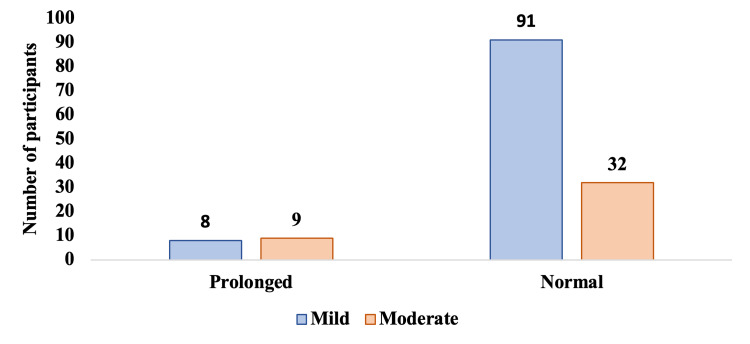
Association between severity and QTc interval among the study participants

As shown in Figure 3, the association between severity and QTc interval indicates that prolonged QTc intervals were significantly higher among study participants with moderate severity (p-value: 0.04).

Table 4 shows the association between severity and QT interval (ms) among the study participants. The QT interval was significantly higher among patients with mild disease compared to those with moderate disease (p-value: <0.01).

**Table 4:** Association between severity and QT interval (ms) ^*^ Mann-Whitney U test

Severity	Mild (n = 99)	Moderate (n = 41)	p-value
Mean QT interval (ms)	374.48 + 37.45	354.27 + 37.35	<0.01*

Table 5 shows the association between severity and QTc dispersion (ms) among the study participants. QTc dispersion was significantly higher among patients with moderate disease compared to mild disease (p-value: <0.01).

**Table 5:** Association between severity and QTc dispersion (ms) ^*^ Mann-Whitney U Test

Severity	Mild (n = 99)	Moderate (n = 41)	p-value
Mean QTc dispersion (ms)	39.39 + 17.09	44.39 + 13.26	<0.01*

Table 6 shows the association between severity and other ECG findings. Sinus tachycardia and RVH with p-pulmonales were significantly higher in moderate severity (p <0.01).

**Table 6:** Association between severity and other ECG findings ^# ^Fisher’s exact test LAFB, left anterior fascicular block; LVH, left ventricular hypertrophy; RBBB, right bundle branch block; RVH, right ventricular hypertrophy

ECG findings	Mild n (%), n = 99	Moderate n (%), n = 41	p-value
Sinus bradycardia	5 (5.5%)	0 (0.0%)	0.32^#^
Sinus tachycardia	9 (9.9%)	12 (29.3%)	<0.01
Sinus arrhythmia	7 (7.7%)	2 (4.9%)	1^#^
LVH	6 (6.6%)	6 (14.6%)	0.1^#^
RVH with p-pulmonale	0 (0.0%)	5 (12.2%)	<0.01
Benign early repolarization	3 (3.3%)	1 (2.4%)	1^#^
LAFB	1 (1.1%)	2 (4.9%)	0.2^#^
RBBB	3 (3.3%)	0 (0.0%)	0.56^#^
Others	3 (3.3%)	0 (0.0%)	0.56^#^
Total	37 (37.3%)	28 (68.3%)	<0.01

**Figure 4:**
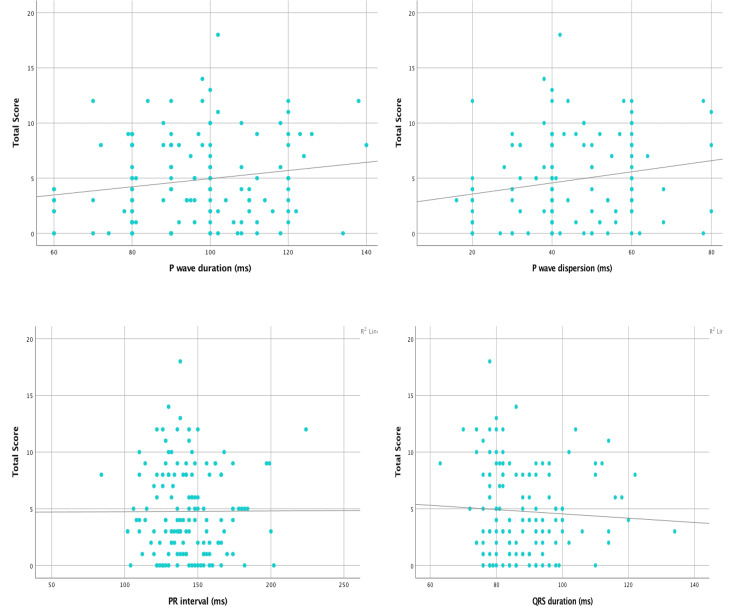
Correlation between ECG indices and disease severity score

**Figure 5:**
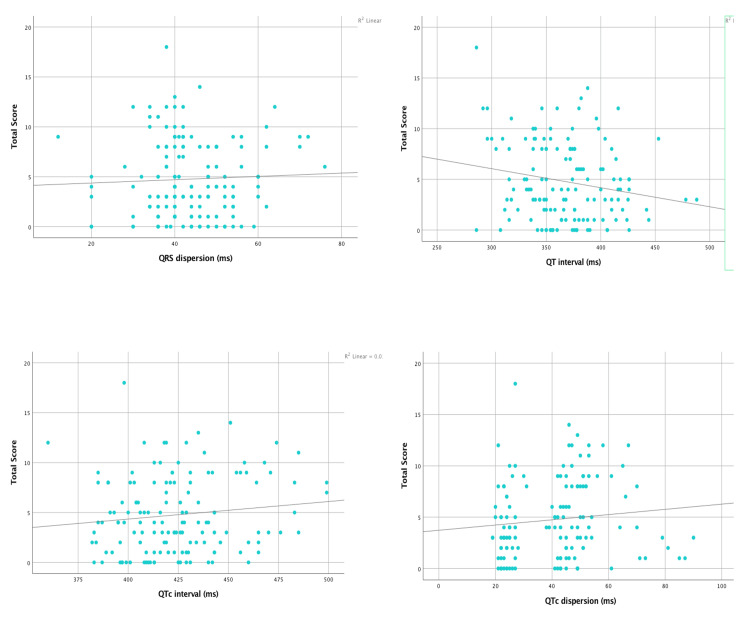
Correlation between ECG indices and disease severity score

Table 7, Figure 4, and Figure 5 show the correlation between ECG indices and disease severity scores. A significant positive correlation was observed between QTc dispersion and severity (r: 0.19; p-value: 0.02). Also, a significant positive correlation was observed between P-wave dispersion and severity (r: 0.17; p-value: 0.04).

**Table 7:** Correlation between ECG indices and disease severity score ^@^ Pearson correlation coefficient ^*^ Significant correlation

ECG interval	r	p-value
P-wave duration (ms)	0.147	0.083
P-wave dispersion (ms)	0.171^*^	0.043
PR interval (ms)	-0.022	0.799
QRS duration (ms)	-0.061	0.473
QRS dispersion (ms)	0.017	0.844
QT interval (ms)	-0.189^*^	.026^@^
QTc interval (ms)	0.117	0.169
QTc dispersion (ms)	0.193^*^	0.023

## Discussion

Out of 140 patients, the mean age of the study participants was 26 ± 6 years. More than half of the cases (80; 57%) fall within the 18-27 age group. Eighty (57.1%) of the participants were males, and the rest, 60 (42.9%), were females. Similar distribution characteristics were found in a study conducted by Mbakamma et al. in 2019 among 53 SCA patients, which showed that 68% of cases and 64% of controls were within the 18-27 age group (range 18-45) [19]. The recurring crises that cause early reporting before the body develops compensatory mechanisms might account for the younger mean population found in multiple studies.

The mean HbS% among the study participants was 66.80 ± 8.02. Also, mean HbS was significantly (p < 0.01) higher among patients with moderate disease (71.28 ± 6.95) compared to mild disease (64.95 ± 7.72). There are hardly any studies comparing the HbS with disease severity, particularly utilizing the Adegoke and Kuti scoring system that we have used in our present study. This could be because of the nonavailability of HPLC reports readily during the study period, multiple HbS values for the same patient interfered with by transfusions, and also due to a nondefinite scoring system for SCA. However, there are studies on fetal hemoglobin’s association with SCA. One such cross-sectional study by Adeodu et al. from Nigeria reported that homozygous sickle cell patients aged 1-15 with moderate disease and potentially fatal complications had significantly lower HbF levels [20].

A total of 99 (70.7%) of the participants had mild disease, 41 (29.3%) had moderate disease, and 0% had severe disease, according to the scoring system used in the study, which is inclusive of all the abovementioned parameters. The most common score was zero, followed by three, one, and eight. The mean severity score among the study participants was 4.76 ± 3.83, with a median (IQR) of 4 (2-8). There was no significant association between the mean age of the study participants or their gender (p = 0.49 and 0.87, respectively). A similar study conducted by Dic-Ijiewere et al. among 120 South Nigerian sickle cell cases in 2021 showed 23%, 65%, and 12% of total cases falling under mild, moderate, and severe disease, respectively, with a mean score of 14.5 ± 4.04 [13]. There was no significant association between severity grade and age groups or gender in the study. The lack of cases in the severe disease category of our study could be due to the exclusion of non-steady-state sickle cell patients who required admission or had recent crises. The exclusion is also justified by the fact that the ECG changes could be affected by confounding factors during the peri-crisis state and might not reveal changes attributable to only the disease severity of an individual.

In our study, the mean heart rate among the study participants was 85 ± 17 beats per minute. The majority of the study participants had a heart rate between 60 and 100 beats per minute. Moreover, five (3.6%) and 22 (15.7%) of the participants had sinus bradycardia and sinus tachycardia, respectively. Most of the study participants had normal axes. Only 11 (7.9%) and five (3.6%) had left and right axis deviations, respectively. The left axis deviation was secondary to LVH or LAFB, while the right axis deviation was secondary to RBBB or RVH. The mean P-wave duration, P-wave dispersion, PR interval, QRS duration, QRS dispersion, QT interval, QTc interval, and QTc dispersion were 94.74 ± 17.72, 43.85 ± 14.32, 142.76 ± 22.29, 88.46 ± 11.83, 43.79 ± 10.5, 368.56 ± 38.41, 423.89 ± 26.86, and 40.86 ± 16.18 ms, respectively.

The QT interval was significantly higher among patients with mild disease compared to those with moderate disease (p-value: <0.01). Although it was statistically significant among the mild disease category, this could also be an apparent association because QT interval is affected by various factors like heart rate, drugs, electrolyte disturbances, myocardial ischemia, hypothermia, etc. However, it was also observed in our study that prolonged QTc interval and QTc dispersion were significantly higher among patients with moderate disease compared to those with mild disease (p-value: 0.04; <0.01, respectively). It is to be noted that QTc is heart rate independent, unlike QT. QTc dispersion also indicates the risk of probable ventricular arrhythmias leading to sudden cardiac deaths and can predict mortality in patients with underlying cardiac dysfunction or even in healthy individuals.

Our study lacked a control population against which the variables mentioned above could be compared. However, similar observations were seen in previous studies. A descriptive cross-sectional study among 60 SCA cases by Oguanobi et al. showed a significant prolongation of dispersion indices (P-wave dispersion, QRS dispersion, and QTc dispersion) among SCA patients and even higher dispersions among SCA patients with pulmonary hypertension [16]. Dic-Ijiewere et al. reported that as the severity of SCA increased, mean P-wave duration, P-wave dispersion, QRS dispersion, QTc interval, and QTc dispersion also increased. For QTc interval and QTc dispersion, these changes were statistically significant (p = 0.003 and p = 0.002, respectively) [13]. These observations raise suspicion about the metabolic cause of these dispersions secondary to hypoxia or toxins (hyperbilirubinemias).

The most common ECG finding in our study was sinus tachycardia (21), followed by LVH (12), sinus arrhythmia (nine), sinus bradycardia (five), RVH with p. pulmonates (five), benign early repolarization (four), LAFB (three), and RBBB (three). Among all these findings, sinus tachycardia and RVH with p-pulmonales were significantly higher with moderate disease severity (p <0.01). Sinus tachycardia could be explained by hypoxia-stimulated chemoreceptors and increased sympathetic activity secondary to anemia. Chronic anemia leads to compensatory LVH. RVH with p-pulmonales was secondary to pulmonary hypertension, the most classic cardiac complication seen in SCA patients. As our study included only steady-state SCA patients, it can be asserted that including severe disease patients or cases with multiple crises would accentuate the findings, uncovering a broader population at risk of various cardiac complications. 

A retrospective study by Sant'Ana PG et al. in Brazil reported that among 50 SCA cases, 21 patients (42%) were hospitalized mainly for painful crises, and 20 patients (40%) received blood transfusions [21].

A case-control study (93 steady-state SCA cases and 90 controls) by Dosunmu et al. in Lagos, Nigeria reported that 73% of the cases had abnormal ECGs, and among these, the common abnormalities observed were LVH, biventricular hypertrophy, and RVH [15]. A retrospective observational study conducted by Ahmed et al. showed significantly (p = 0.01) higher pulmonary arterial systolic pressure (43.2 ± 0.5) among patients with low Hb (<8 g/dl) as compared to patients with Hb >/= 8 g/dl [12].

Our study also determined if there was any correlation between the various ECG changes and disease severity. Among all the parameters studied, a significant positive correlation was observed between QTc dispersion and severity (r: 0.19; p-value: 0.02). Also, a significant positive correlation was observed between P-wave dispersion and severity (r = 0.17; p = 0.04). This positive correlation also reiterates the fact that as the disease severity progresses, the risk of atrial and ventricular arrhythmias also increases, as indicated by the P-wave and QTc dispersion, respectively. Although there are studies available on determining different ECG changes in SCA patients, very few studies have correlated these changes with disease severity. This could largely be due to the lack of a universal scoring system for SCA. One such study conducted by Dic-Ijiewere et al. showed that there was a significant positive correlation between the SCA severity scores and P-wave duration, P-wave dispersion, QRS dispersion, QTc interval, and QTc dispersion (r: 0.32; p: 0.01; r: 0.27; p: 0.03; r: 0.29; p: 0.02; r: 0.33; p: 0.01; and r: 0.34; p: 0.007, respectively), suggesting a higher risk of left atrial abnormalities, intraventricular conduction abnormalities, increased risk of arrhythmias, and sudden death as disease severity progresses [13].

Limitations

Only steady-state sickle patients from OPD were included in the study, not the ones with active or recent crises. As it was a cross-sectional study, there was no follow-up to study the ECG changes and their relation to disease severity. Consequently, the validity of the changes observed and the correlation studied cannot be determined due to the uncertainty of the persistence of outcomes in the long term. Patients were not followed up with a 2D echo for confirmation of the changes studied. The study lacked a control group against which the outcomes could be compared. As the study was conducted in a tertiary care center, the outcomes cannot be extrapolated to the much broader sickle cell population, especially where SCD is endemic in a state like Chhattisgarh.

## Conclusions

Among all the ECG parameters studied, QTc dispersion, prolonged QTc interval, sinus tachycardia, and RVH with p-pulmonales were significantly higher in the moderate disease category. A significant positive correlation was observed for P and QTc dispersions with disease severity. ECG is a readily and widely accessible investigation that can be used to screen all SCA patients for early recognition of various underlying cardiac complications.
